# Association of the Advanced Lung Cancer Inflammation Index (ALI) and Gustave Roussy Immune (GRIm) score with immune checkpoint inhibitor efficacy in patients with gastrointestinal and lung cancer

**DOI:** 10.1186/s12885-024-12149-1

**Published:** 2024-04-08

**Authors:** Hao Jiang, Borui Li, Min Wu, Qimei Wang, Yijin Li

**Affiliations:** 1 Department of General Surgery, Taizhou Central Hospital (Taizhou University, Hospital), Taizhou, China; 2 Department of Urologic Surgery, Cancer Hospital of China Medical University (Liaoning Cancer Hospital & Institute), Shenyang, China; 3 Department of Oncology, The Third People’s Hospital of Honghe Prefecture, Gejiu, China; 4 Department of Colorectal and Anorectal Surgery, Hunan Hospital of Integrated Tradmonal Chinese and Western Medicine (Hunan Academy of Traditional Chinese Medicine Affiliated Hospital), Changsha, China; 5 Hunan Academy of Traditional Chinese Medicine, Changsha, China

**Keywords:** Advanced lung cancer inflammation index, Gustave Roussy Immune score, Cancers, Immune checkpoint inhibitor, Hepatocellular carcinoma modified Gustave Roussy Immune score

## Abstract

**Objective:**

This study aimed to conduct a comprehensive analysis, evaluating the prognostic significance of the baseline Advanced Lung Cancer Inflammation Index (ALI) and Gustave Roussy Immune (GRIm) Score in patients undergoing immune checkpoint inhibitor (ICI) therapy.

**Methods:**

A comprehensive search was performed across various databases, including PubMed, the Cochrane Library, EMBASE, and Google Scholar, until October 21, 2023, to compile relevant articles for analysis. The investigation encompassed diverse clinical outcomes, including overall survival (OS) and progression-free survival (PFS).

**Results:**

This analysis included a total of 15 articles, comprising 19 studies involving 3335 patients. Among the 19 studies, nine studies focused on NSCLC, and six studies were conducted on HCC. Pooled results revealed that patients with elevated ALI levels experienced prolonged OS (HR: 0.51, 95% CI: 0.37–0.70, *p* < 0.001) and extended PFS (HR: 0.61, 95% CI: 0.52–0.72, *p* < 0.001). Furthermore, a GRIm score > 1 was associated with reduced OS (HR: 2.07, 95% CI: 1.47–2.92, *p* < 0.001) and diminished PFS (HR: 1.78, 95% CI: 1.35–2.34, *p* < 0.001) in cancer patients receiving ICIs. Subgroup analysis indicated that ALI cutoff values of 18 exhibited enhanced predictive potential. Additionally, for HCC patients, those with HCC-GRIm score > 2 showed a substantially decreased risk of mortality compared to individuals with HCC-GRIm score ≤ 2 (HR: 2.63, 95% CI: 1.89–3.65, *p* < 0.001).

**Conclusion:**

The ALI and GRIm score served as dependable prognostic indicators for patients undergoing ICI therapy in the context of cancer treatment.

**Supplementary Information:**

The online version contains supplementary material available at 10.1186/s12885-024-12149-1.

## Introduction

In recent years, immune checkpoint inhibitors (ICIs) that target the PD-1/PD-L1 axis have become an integral part of clinical practice [1, 2]. They have revolutionized the treatment of various cancers, including non-small cell lung cancer (NSCLC), head and neck squamous cell carcinoma (HNSCC), renal cell carcinoma (RCC), urothelial cancer (UC), melanoma, and hepatocellular carcinoma (HCC) [3, 4]. While ICI therapy has demonstrated remarkable response rates and long-term survival in advanced cancer patients, its high cost presents economic challenges. Moreover, not all patients benefit equally from immunotherapy [5]. A comprehensive analysis of 262 patients with different malignancies revealed an overall objective response rate (ORR) of 29% and a long-term survivor rate (i.e., longer than 2 years) of 11.8% [6]. It is worth noting that the immune-related adverse effects of ICI therapy can lead to severe and, in some cases, fatal consequences [7].

In recent years, there has been an increasing emphasis on the early identification of non-responsive individuals to ICI therapy in cancer treatment to avoid ineffective treatments and reduce the risk of adverse effects [8, 9]. Numerous predictive biomarkers have been studied for their association with the ICI response, such as intratumoral PD-L1 expression, tumor mutational burden, T-cell infiltration metrics, and the use of antibiotics and acid suppressants [5, 10]. Nevertheless, establishing consistent criteria for quantifying these markers remains a challenge. At present, the only marker that has received regulatory approval as a companion diagnostic for ICI treatment is the detection of intratumoral PD-L1 [11, 12]. However, the predictive value of PD-L1 expression has not been clarified and recently published meta-analyses come to different conclusions [13, 14]. Therefore, it is crucial to identify novel prognostic biomarkers that can enhance the outcomes of cancer patients undergoing ICI therapy.

Blood tests offer several advantages, including clinical applicability, simplicity, affordability, and the ability to provide objective analysis from virtually anywhere. Cancer tumorigenesis and metastasis are associated with systemic inflammation and malnutrition, and there is a mounting body of evidence emphasizing the pivotal role of inflammation in cancer progression [15]. Nutritional and inflammatory indicators, with the most extensively studied ones being albumin and neutrophil-to-lymphocyte ratio (NLR), have demonstrated the ability to predict the efficacy of ICI therapy in oncology patients [8, 16]. In 2013, Jafri et al. incorporated NLR, albumin, and body mass index (BMI) into a model, and it was revealed that advanced lung cancer inflammation index (ALI) might help predict survival outcomes in various tumors [17, 18]. The Gustave Roussy Immune (GRIm) score, a prognostic tool, combines three key biomarkers: NLR, albumin, and lactate dehydrogenase (LDH). By combining these factors, patients can be categorized into high-risk and low-risk groups, with those with a GRIm score > 1 considered to have a high score [19–21]. The predictive value of the GRIm score has been extensively studied in patients with advanced NSCLC who have received various treatments, including cytotoxic chemotherapy, epidermal growth factor receptor tyrosine kinase inhibitors (EGFR-TKIs), or second-line immunotherapy [19–21]. Besides, an improved GRIm score (hepatocellular carcinoma modified Gustave Roussy Immune Score, HCC-GRIm score) was proposed by Li et al. [22]. Compared to the original GRIm-Score, they discovered that the HCC-GRIm-Score had higher predictive power in identifying the HCC patients potentially benefiting from ICIs therapy [22].

The correlation between ALI levels, GRIm score, and the prognosis of cancer patients receiving ICI therapy has yielded conflicting results, and a comprehensive meta-analysis on this topic is currently lacking. Hence, the aim of this study was to systematically evaluate the predictive significance of ALI levels and GRIm score in cancer patients undergoing ICI therapy. The results of this investigation possess the capability to significantly contribute to the advancement of ICIs. This contribution lies in facilitating the delivery of precise, cost-effective treatments and minimizing adverse effects.

## Methods

### Literature search strategies

In adherence to the PRISMA statement [23], the analysis in this study followed a meticulous approach. An extensive literature search was conducted on October 21, 2023, across reputable databases, including PubMed, EMBASE, and the Cochrane Library. We used a variety of search terms, including both MeSH terms and keywords, to identify relevant studies. These terms included the following: “Immune Checkpoint Inhibitors [MeSH]”, “PD-1 Inhibitors”, “PD-L1 Inhibitors”, “CTLA-4 Inhibitors”, “Pembrolizumab”, “Nivolumab”, “Atezolizumab”, “Ipilimumab”, “Avelumab”, “Tremelimumab”, “Durvalumab”, “Cemiplimab”, “advanced lung cancer inflammation index”, “Gustave Roussy Immune Score”, and “GRIm”. A comprehensive description of the search strategies employed can be found in Supplementary material 1. Additionally, a thorough exploration of grey literature was performed using Google Scholar, while the reference lists of eligible studies were manually screened to ensure inclusiveness.

### Inclusion and exclusion criteria

In this study, we rigorously selected research articles that met specific criteria. These criteria included patients diagnosed with cancer who received treatment with ICIs and the evaluation of the ALI or GRIm score as a prognostic factor. Furthermore, the articles were required to report on at least one of the following outcomes: overall survival (OS) and progression-free survival (PFS). To maintain the focus of our analysis, conference abstracts, comments, and case reports were excluded [24]. When there were studies with overlapping patient cohorts, priority was given to those with the most comprehensive data and robust methodology [25].

### Data extraction and quality assessment

In this study, we gathered a variety of information from the selected articles, encompassing author names, publication year, study duration and location, treatment drugs, cancer type, sample size, patient demographics (age and gender), and pertinent cut-off values and outcomes. Our focus was particularly directed toward acquiring data from multivariate analyses, which offer more comprehensive insights compared to univariate analyses. To assess the quality of observational studies, we employed the Newcastle-Ottawa Scale (NOS) and categorized studies with a NOS score of 6 or higher as high-quality literature [8]. The above process has been independently completed and cross-checked by two authors, with senior authors consulted on any disputes.

### Statistical methods

The meta-analyses were executed utilizing Stata 15.0. Heterogeneity was evaluated employing the chi-squared test. In instances where studies displayed a *p*-value < 0.1 or an I^2^ statistic exceeding 50%, the application of a random effects model was warranted; conversely, a fixed effects model was applied. Examination for potential publication bias involved both Egger’s [26] and Begg’s tests [27]. In the event of bias detection, the “trim and fill” approach was implemented to gauge its impact on the aggregated outcomes [28]. Furthermore, to assess the resilience of the findings, a sensitivity analysis was performed through the systematic exclusion of each individual study.

## Results

### Characteristics of studies

**Fig. 1:**
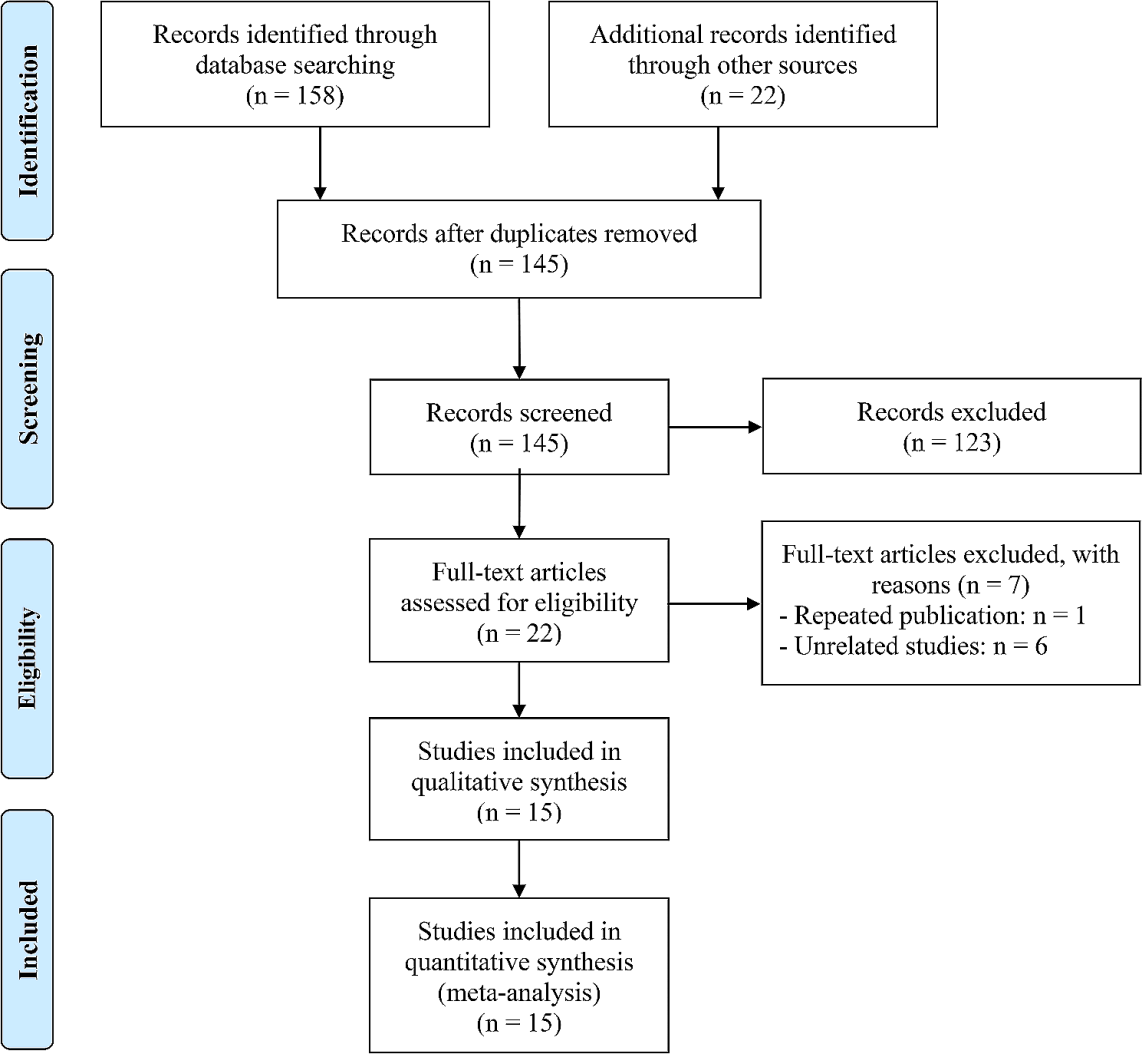
The flow diagram for identifying eligible studies

Following the removal of duplicate studies and initial screening of titles and abstracts, we proceeded to evaluate the full texts of 22 articles. Among them, 15 articles with 19 studies met the eligibility criteria, resulting in a total of 3335 patients [19, 22, 29–41]. The study selection process is presented in Fig. 1, following the guidelines of the PRISMA flowchart. For a comprehensive understanding of the characteristics of the included studies, Table 1 provides an overview. To assess the risk of bias, we utilized the Newcastle-Ottawa Scale (NOS), which indicated a low risk of bias across all included studies, with score ranging from 6 to 8. Among the 19 studies, nine studies focused on NSCLC, and six studies were conducted on HCC. There was one study each for GC and SCLC. 11 studies investigated the predictive value of ALI, whereas seven studies evaluated the predictive significance of GRIm. Additionally, three studies examined the predictive value of the HCC-GRIm score.

**Table 1:** Main characteristics of the studies included

Study	Study design	Study period	Study region	Cancer type	ICI treatment	Sample size	Age (years)	Gender (male/female)	TNM stage (I/II/III/IV)	Outcomes	NOS Score
Li. Q et al. 2023 (D)	R	02/2018-02/2019	China	HCC	Anti-PD-1 inhibitors	98	52^d^	66/32	-	ALI (OS, PFS)	7
Li. Q et al. 2023 (V)	R	04/2019-04/2020	China	HCC	Anti-PD-1 inhibitors	52	-	44/8	-	ALI (OS)	6
Yamaguchi et al. 2023	R	12/2020-03/2022	Japan	NSCLC	Nivolumab plus ipilimumab	101	31/70^f^	83/18	-	ALI (OS, PFS)	7
Liu et al. 2023	R	01/2019-12/2021	China	HCC	ICIs	151	57 ± 9.1	124/27	4/55/70/22	ALI (OS, PFS)	7
Hatanaka et al. 2023	R	09/2020-01/2022	Japan	HCC	Atezolizumab and bevacizumab	405	74 (68–79)^b^	328/77	-	HCC-GRIm-Score (OS)	8
Nakazawa et al. 2023	R	10/2017-12/2018	Japan	GC	Nivolumab	58	66^d^	45/13	-	GRIm (OS, PFS)	6
Minichsdorfer et al. 2023	R	01/2015-11/2016	Austria	Cancer	Pembrolizumab, Nivolumab	114	60 (22–88)^a^	74/40	-	GRIm (OS, PFS)	7
Holtzman et al. 2022 (A)	R	06/2016-12/2020	Israeli	NSCLC	Pembrolizumab	302	70 (36–97)^a^	200/102	-	ALI (OS)	7
Holtzman et al. 2022 (B)	R	06/2016-12/2020	Israeli	NSCLC	Pembrolizumab	121	66 (35–87)^a^	74/47	-	ALI (OS)	7
Qi et al. 2021	R	-	China	SCLC	Atezolizumab	53	26/27^e^	34/19	-	ALI (OS)	6
Mountzios et al. 2021 (A)	R	-	Greece, Germany	NSCLC	Anti-PD-L1 inhibitors	460	67 ± 10	324/136	-	ALI (OS, PFS)	8
Mountzios et al. 2021 (B)	R	-	Greece, Germany	NSCLC	Anti-PD-L1 inhibitors	212	67 ± 10	137/75	-	ALI (OS, PFS)	7
Li, Y et al. 2021 (T)	R	01/2018-12/2019	China	HCC	Pembrolizumab, Nivolumab, Toripalimab, Sintilimab, Tislelizumab, Camrelizumab	181	32/129^c^	157/24	-	GRIm (OS), HCC-GRIm score (OS)	6
Li, Y et al. 2021 (V)	R	01/2020-09/2020	China	HCC	Pembrolizumab, Nivolumab, Toripalimab, Sintilimab, Tislelizumab, Camrelizumab	80	25/55^c^	45/35	-	GRIm (OS), HCC-GRIm score (OS)	7
Lenci et al. 2021	R	07/2017-07/2020	Italy	NSCLC	Pembrolizumab	135	71 (44–91)^a^	84/51	-	GRIm (OS, PFS)	7
Al Darazi et al. 2020	R	02/2015-12/2018	French	Cancer	ICIs	259	63 (18–83)^a^	169/90	-	GRIm (OS)	7
Adachi et al. 2020	R	12/2015-12/2018	Japan	NSCLC	Nivolumab	296	70 (64-76)^b^	206/90	-	ALI (OS, PFS)	8
Minami et al. 2019	R	12/2015-10/2018	Japan	NSCLC	Nivolumab, Pembrolizumab, Atezolizumab	76	-	49/27	-	GRIm (OS, PFS)	7
Shiroyama et al. 2018	R	12/2015-05/2016	Japan	NSCLC	Nivolumab	201	68 (27–87)^a^	135/66	-	ALI (PFS)	7

^a^medians (ranges); ^b^medians (interquartile range); ^c^≥ 60 vs. < 60; ^d^mean/median; ^e^≥ 65 vs. < 65; ^f^≥ 75 vs. < 75; R, retrospective cohort study; HCC, hepatocellular carcinoma; GC, gastric cancer; NSCLC, non-small-cell lung cancer; SCLC, small-cell lung cancer; PD-L1, programmed cell death 1 ligand 1; PD-1, programmed cell death protein 1; ICI, immune checkpoint inhibitor; ALI, advanced lung cancer inflammation index; GRIm, Gustave Roussy Immune Score; HCC-GRIm, hepatocellular carcinoma modified Gustave Roussy Immune Score; OS, overall survival; PFS, progression-free survival

### Baseline ALI levels and OS

**Fig. 2:**
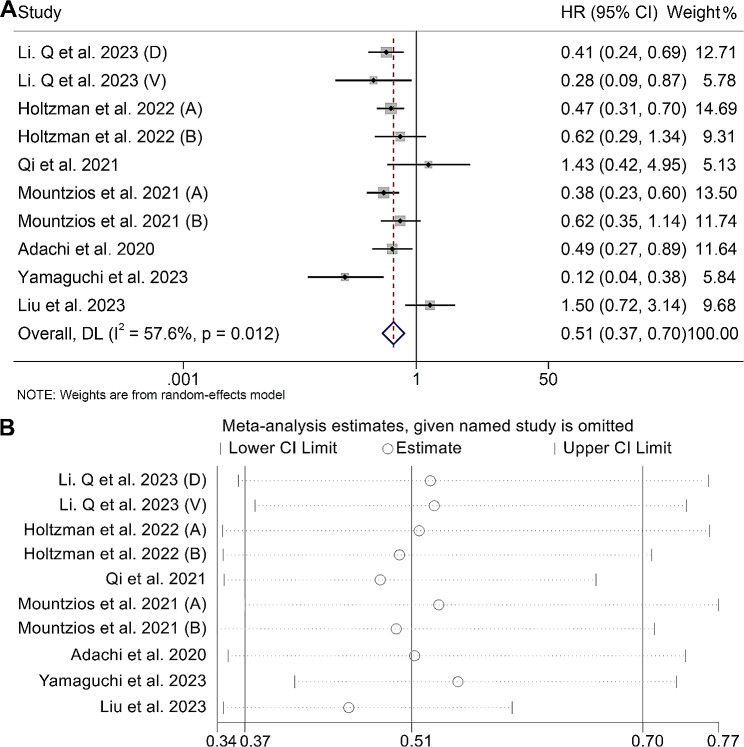
(**A**) Forest plots of the relationship between advanced lung cancer inflammation index and overall survival (heterogeneity index I^2^ = 57.6%, *p* = 0.012; HR: 0.51, 95% CI: 0.37–0.70, *p* < 0.001). (**B**) Sensitivity analysis of the association between advanced lung cancer inflammation index and overall survival. HR, hazard ratio; CL, confidence interval

By analyzing data from a total of ten studies involving 1846 patients, we investigate the association between ALI levels and OS in cancer patients treated with ICIs. Notably, the results exhibited significant heterogeneity (I^2^ = 57.6%, *p* = 0.012), allowing us to employ a random-effects model for estimating the pooled HR. The findings demonstrated a significant correlation, indicating that higher ALI levels were associated with improved OS (HR: 0.51, 95% CI: 0.37–0.70, *p* < 0.001), as illustrated in Fig. 2A.

To evaluate the reliability of the outcomes, a sensitivity analysis was executed by systematically excluding each individual study and scrutinizing its impact on the overall findings. Notably, the exclusion of any specific study did not exert a substantial effect on the aggregated hazard ratio (HR) for overall survival (OS). Precisely, HR estimates for OS ranged from 0.45 (95% CI: 0.35–0.59) upon excluding the investigation conducted by Liu et al. 2023 to 0.55 (95% CI: 0.41–0.73) upon excluding the study conducted by Yamaguchi et al. 2023 (Fig. 2B). These observations underscore the robustness and consistency of our analysis. To gauge potential publication bias in our meta-analysis, we conducted both Begg’s and Egger’s tests. The outcomes revealed no statistically significant indications of publication bias (Egger’s test: *p* = 0.822, Begg’s test: *p* = 0.592).

### Baseline ALI levels and PFS

**Fig. 3:**
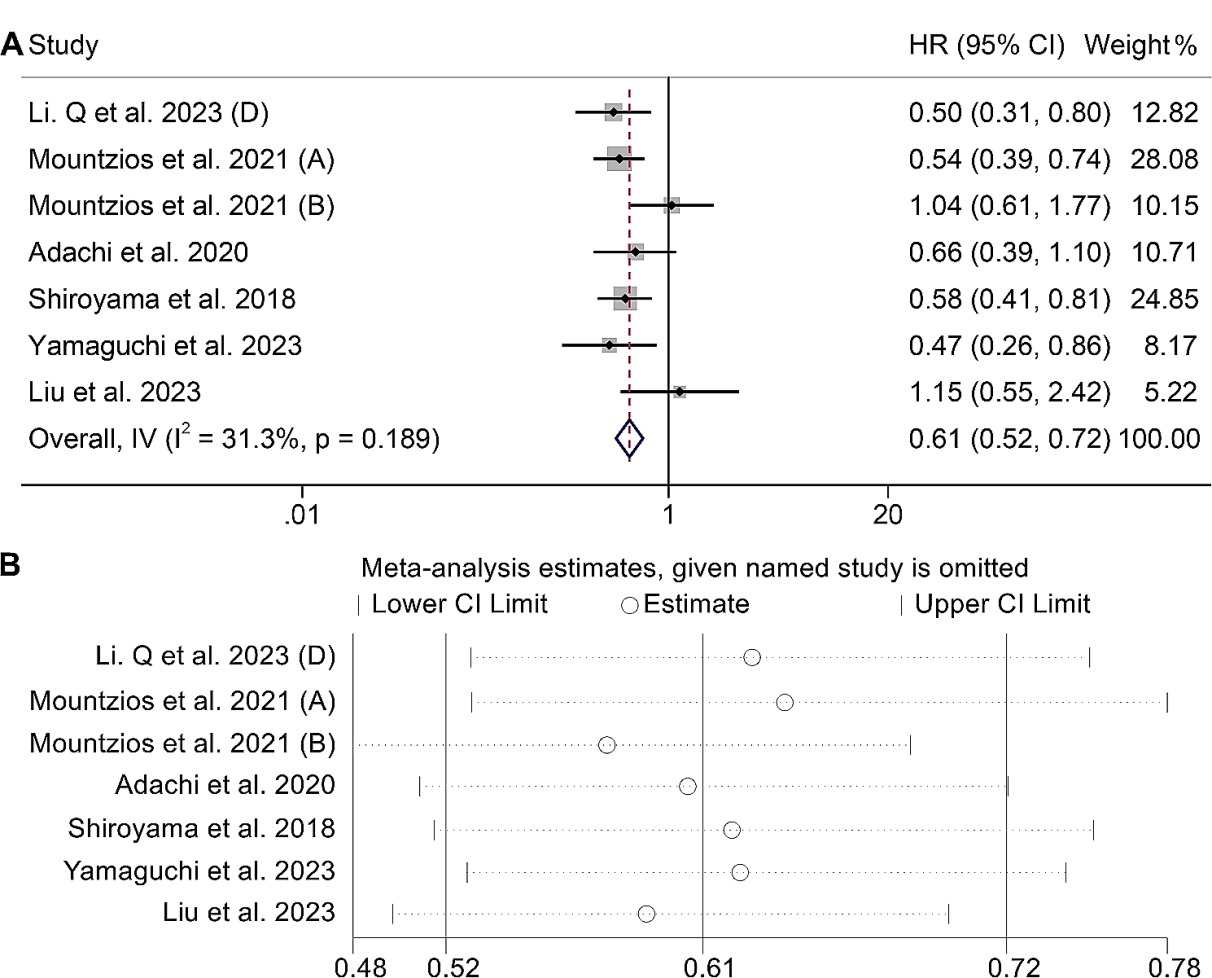
(**A**) Forest plots of the relationship between advanced lung cancer inflammation index and progression-free survival (heterogeneity index I^2^ = 31.3%, *p* = 0.189; HR: 0.61, 95% CI: 0.52–0.72, *p* < 0.001). (**B**) Sensitivity analysis of the association between advanced lung cancer inflammation index and progression-free survival. HR, hazard ratio; CL, confidence interval

In our analysis, we also examined the association between ALI and PFS in cancer patients treated with ICIs. A total of seven studies encompassing 1519 patients were included in our investigation. Our findings revealed that patients with high ALI levels exhibited a significantly reduced risk of disease progression (HR: 0.61, 95% CI: 0.52–0.72, *p* < 0.001, Fig. 3A). Notably, no substantial heterogeneity was observed among the studies (I^2^ = 31.3%, *p* = 0.189), thus the fixed-effects model was used.

Begg’s and Egger’s tests confirmed that there was no publication bias in the above results (Egger’s test: *p* = 0.204, Begg’s test: *p* = 0.133). The sensitivity analysis revealed that the exclusion of any individual study did not have a significant impact on the overall results for PFS. The range of HR values for PFS varied from 0.58 (95% CI: 0.48–0.69) when excluding the study by Mountzios et al. 2021 (B) to 0.65 (95% CI: 0.51–0.85) after excluding Mountzios et al. 2021 (A), as shown in Fig. 3B. This analysis indicates that the overall findings regarding PFS remain robust and unaffected by the removal of any specific study.

### Baseline GRIm score and OS

**Fig. 4:**
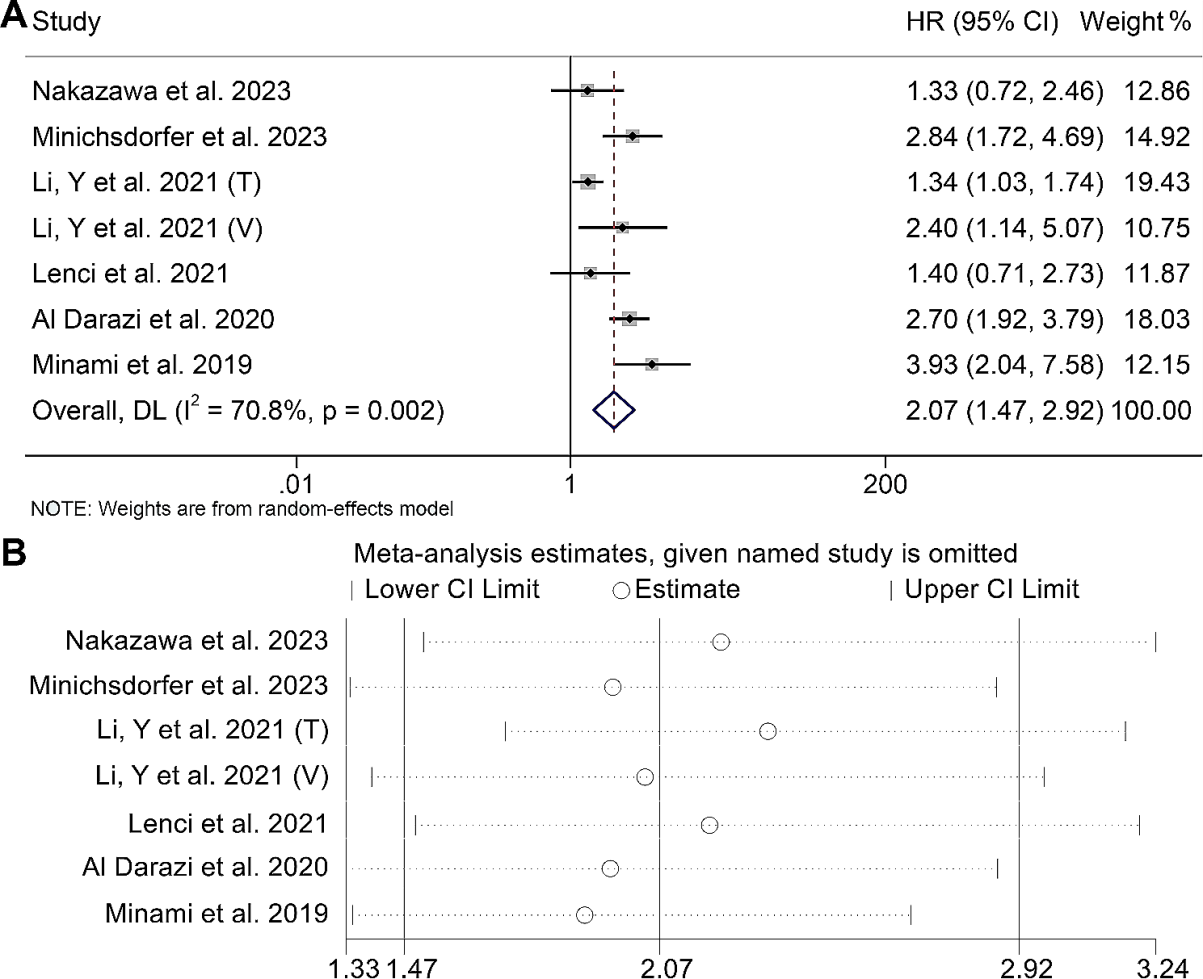
(**A**) Forest plots of the relationship between Gustave Roussy Immune Score and overall survival (I^2^ = 70.8%, *p* = 0.002; HR: 2.07, 95% CI: 1.47–2.92, *p* < 0.001). (**B**) Sensitivity analysis of the association between the Gustave Roussy Immune Score and overall survival. HR, hazard ratio; CL, confidence interval

Using data from seven studies comprising 903 patients, we investigated the association between GRIm score and OS in patients with cancer treated with ICIs. Our findings demonstrated that patients with a GRIm score > 1 were associated with a 107% increased risk of death (HR: 2.07, 95% CI: 1.47–2.92, *p* < 0.001, Fig. 4A) than those with a GRIm score ≤ 1. Considering the presence of significant heterogeneity (I^2^ = 70.8%, *p* = 0.002), we employed a random-effects model to account for the variability among the included studies.

The results of Begg’s and Egger’s tests provided further confirmation that no publication bias was present in the aforementioned findings (Egger’s test: *p* = 0.405, Begg’s test: *p* = 1.000). The sensitivity analysis confirmed that the exclusion of any individual study did not significantly alter the overall results. The range of HR values for OS varied from 1.96 (95% CI: 1.33–2.87) after excluding Al Darazi et al. 2020 to 2.22 (95% CI: 1.52–3.24) after excluding Nakazawa et al. 2023 (Fig. 4B).

### Baseline GRIm score and PFS

**Fig. 5:**
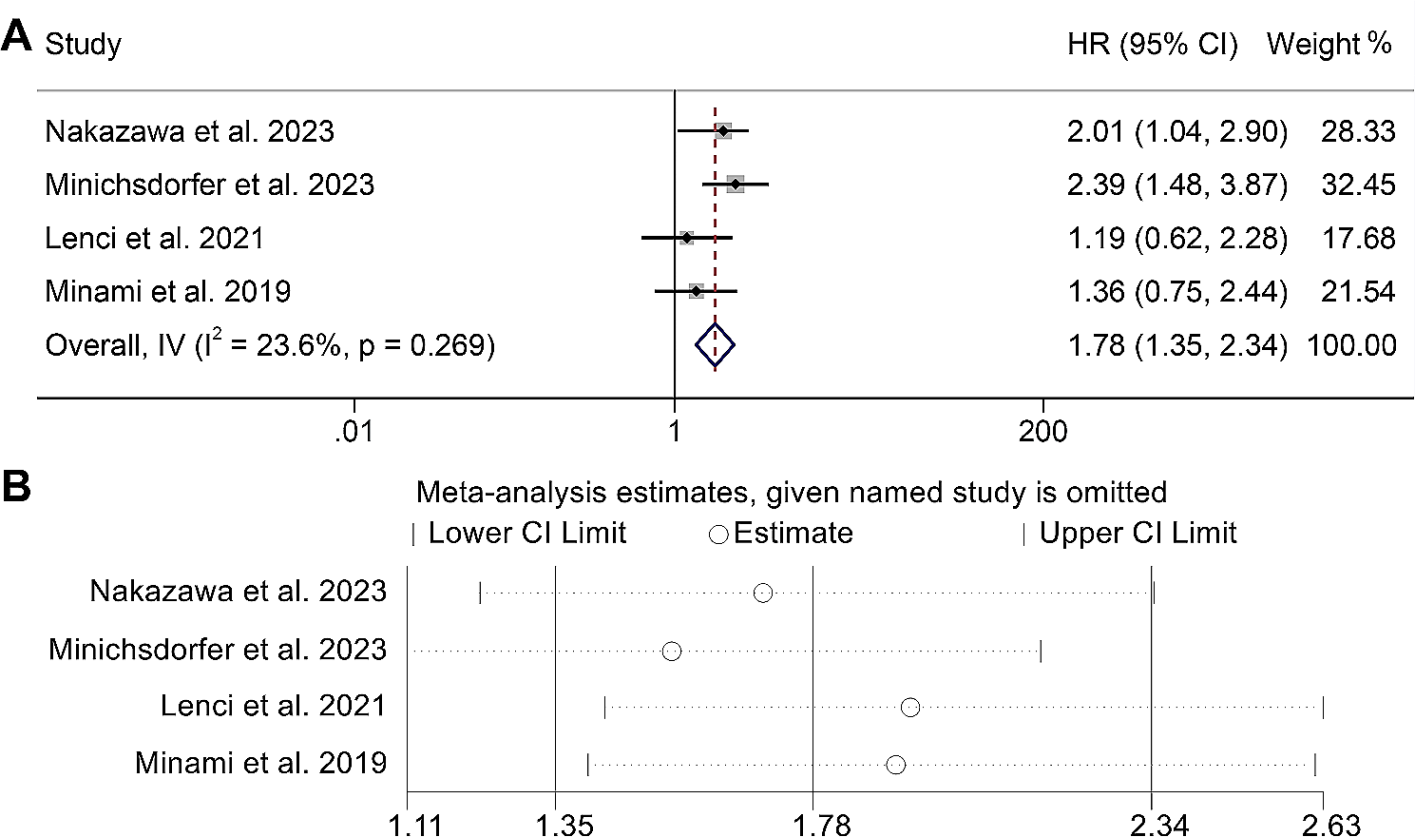
(**A**) Forest plots of the relationship between Gustave Roussy Immune Score and progression-free survival (I^2^ = 23.6%, *p* = 0.269; HR: 1.78, 95% CI: 1.35–2.34, *p* < 0.001). (**B**) Sensitivity analysis of the association between the Gustave Roussy Immune Score and progression-free survival. HR, hazard ratio; CL, confidence interval

Our study also examined the relationship between GRIm score and PFS in cancer patients treated with ICIs by analyzing data from four studies comprising 383 participants. There was no significant heterogeneity (I^2^ = 23.6%, *p* = 0.269), and a fixed-effects model was used to estimate the pooled HR. As shown in Fig. 5A, the findings revealed a GRIm score > 1 related to shorter PFS in cancer patients (HR: 1.78, 95% CI: 1.35–2.34, *p* < 0.001). Sensitivity analysis confirmed that the results were stable and reliable (Fig. 5B). However, we found a publication bias in the above results (Egger’s test: *p* = 0.012, Begg’s test: *p* = 0.089). To address this bias, we employed the trim and fill method to estimate the potential number of missing studies. However, the inclusion of these hypothetical missing studies did not result in any significant changes to the pooled HR (HR: 1.72, 95% CI: 1.36–2.34, *p* < 0.001). Therefore, it can be inferred that, despite the presence of publication bias, the overall findings remain largely unaffected and maintain their robustness.

### Baseline HCC-GRIm score and OS

**Fig. 6:**
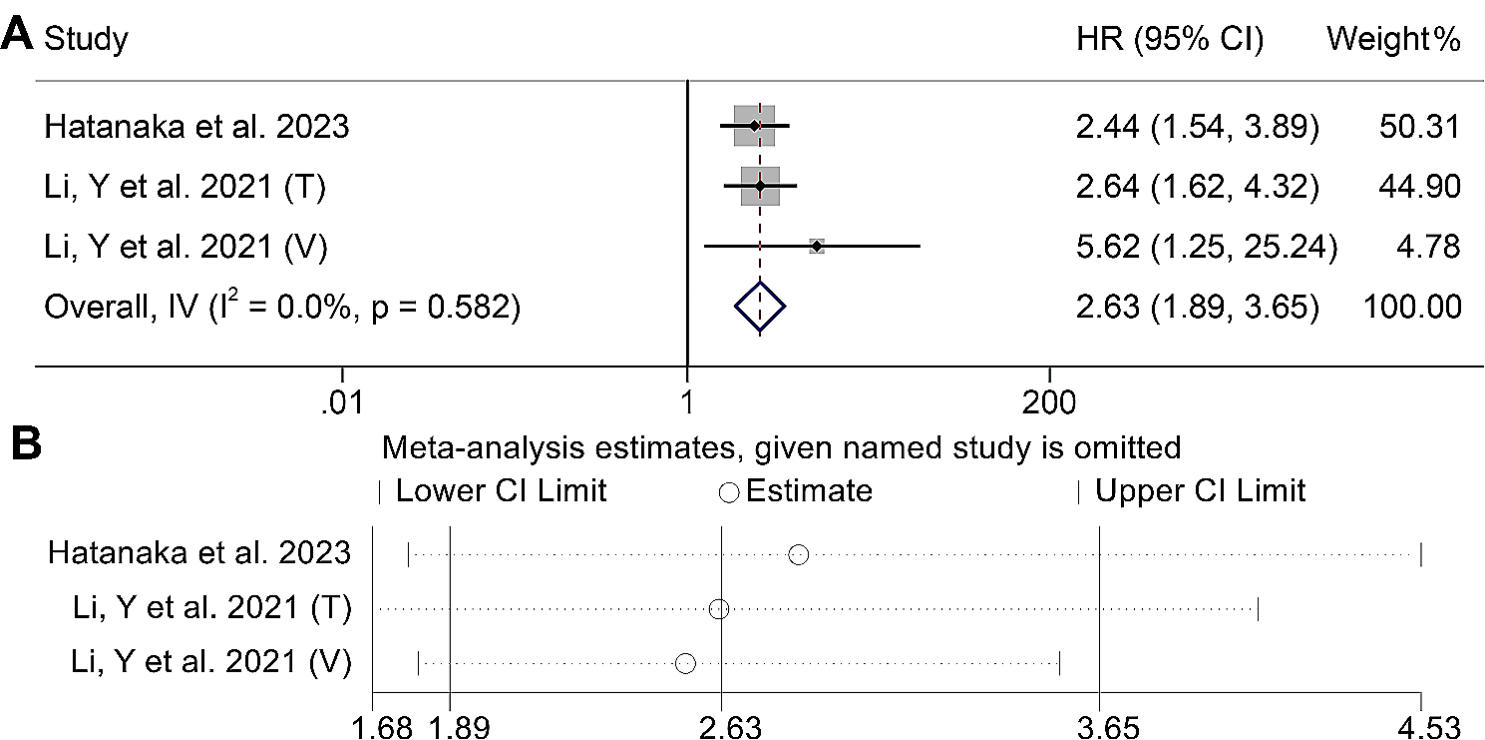
(**A**) Forest plots of the relationship between hepatocellular carcinoma-modified Gustave Roussy Immune Score and overall survival (I^2^ = 0.0%, *p* = 0.582, HR: 2.63, 95% CI: 1.89–3.65, *p* < 0.001). (**B**) Sensitivity analysis of the association between hepatocellular carcinoma-modified Gustave Roussy Immune Score and overall survival. HR, hazard ratio; CL, confidence interval

We also analyzed the relationship between the HCC-GRIm score and OS of HCC patients treated with ICIs using prognostic data from three cohorts of 666 individuals. Our findings indicate that patients with an HCC-GRIm score > 2 face a significantly elevated risk of mortality compared to those with an HCC-GRIm score ≤ 2 (I^2^ = 0.0%, *p* = 0.582, HR: 2.63, 95% CI: 1.89–3.65, *p* < 0.001, Fig. 6A). Further validation was provided by the results of Begg’s and Egger’s tests, which demonstrated the absence of publication bias in these findings (Egger’s test: *p* = 0.103, Begg’s test: *p* = 0.296). Sensitivity analysis confirmed the stability and reliability of the results (Fig. 6B).

### Subgroup analysis

**Fig. 7:**
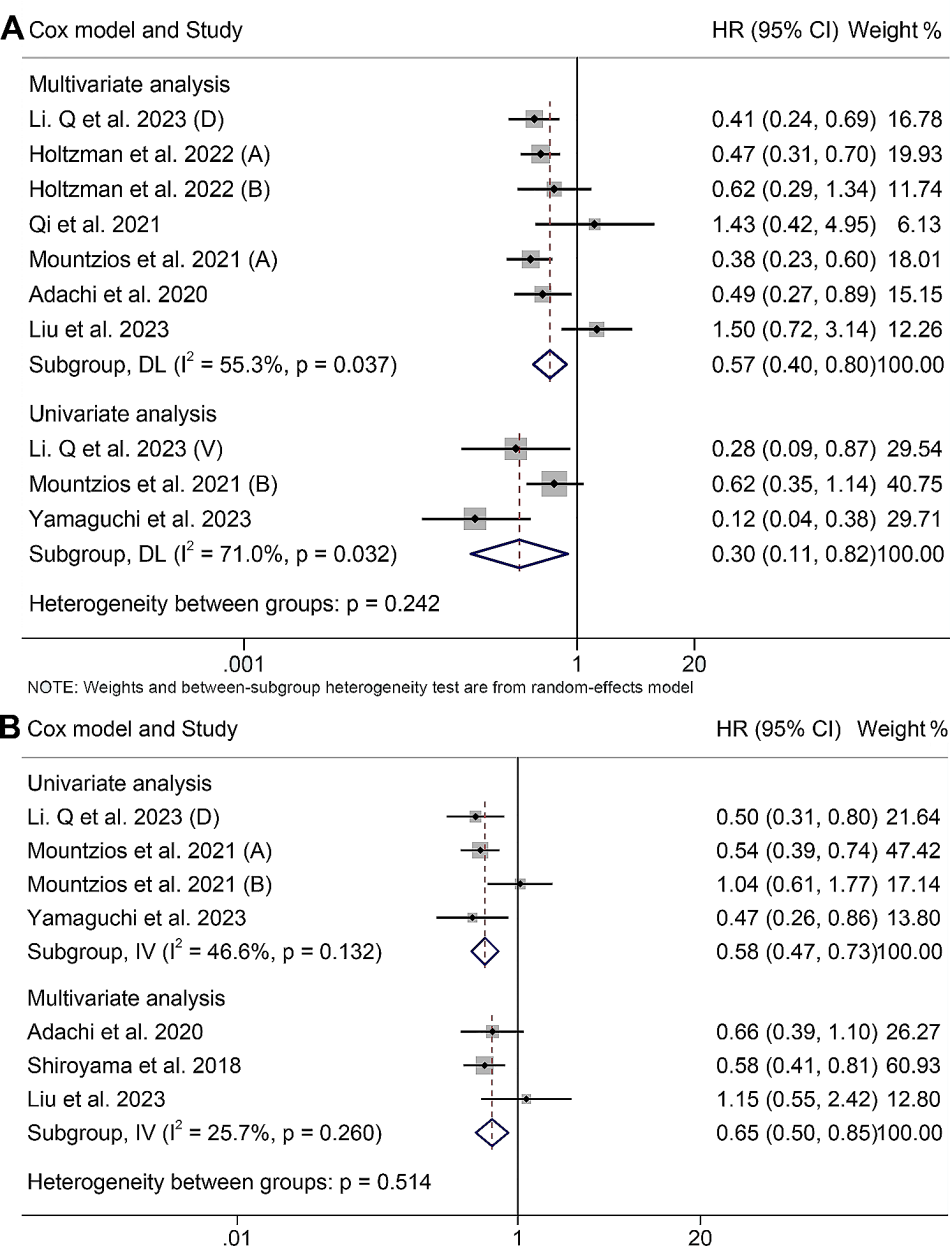
Subgroup analysis of the relationship between advanced lung cancer inflammation index and overall survival (**A**) and progression-free survival (**B**) based on the Cox model. HR, hazard ratio; CL, confidence interval

We first performed a subgroup analysis according to the Cox models. We found that high ALI levels were associated with longer OS and PFS in both univariate (OS, HR: 0.30, 95% CI: 0.11–0.82, *p* = 0.019; PFS, HR: 0.58, 95% CI: 0.47–0.73, *p* < 0.001) and multivariate analyses (OS, HR: 0.57, 95% CI: 0.40–0.80, *p* = 0.001; PFS, HR: 0.65, 95% CI: 0.50–0.85, *p* = 0.002) (Fig. 7A and B). Subgroup analysis was then performed according to the different cut-off values. When the ALI was bounded by 18, higher ALI was associated with better OS (HR: 0.48, 95% CI: 0.38–0.611, *p* < 0.001) and PFS (HR: 0.62, 95% CI: 0.51–0.76, *p* < 0.001) (Figure S1A and S1B). And when ALI was cut off by other values, the above relationship did not hold (Figure S1A and S1B).

## Discussion

Our study aimed to investigate the prognostic implications of the ALI and GRIm score in cancer patients receiving ICI therapy. Through a comprehensive meta-analysis of relevant studies, we established a strong correlation between higher ALI levels, a lower GRIm score, and improved OS and PFS. Furthermore, subgroup analysis revealed that ALI cutoff values of 18 demonstrated higher predictive potential.

The systemic inflammation observed in cancer patients stems from various factors, including cancer itself, the release of inflammatory mediators by leukocytes, and tissue inflammation triggered by tumor growth or invasion [42–44]. Inflammatory markers have proven to be valuable predictors because systemic inflammatory responses contribute to cancer progression, invasion, and metastasis [45–47]. In patients undergoing ICI therapy, cytokines and chemokines produced by neutrophils can promote angiogenesis and remodeling of the extracellular matrix [48, 49]. This, in turn, creates a favorable microenvironment for cancer growth and influences the effectiveness of ICIs [50, 51]. Additionally, lymphocytes play a critical role in antitumor immune responses by recognizing cancer cell antigens [52], and these biomarkers may reflect the immune status of patients and their response to ICIs [53].

Several studies have demonstrated the significance of the NLR as a predictive marker for therapeutic response to ICIs in various cancers [54, 55]. Elevated levels of proinflammatory cytokines, such as osteopontin and interleukin-6, have been associated with poor outcomes in cancer [56, 57]. Furthermore, a higher NLR value is often correlated with increased proinflammatory cytokine levels [58, 59]. Furthermore, higher NLR values contribute to increased infiltration of macrophages in the tumor microenvironment, leading to resistance to ICIs [60].

Historical investigations have established a correlation between malnutrition and an unfavorable tumor prognosis [43, 61]. Although diminished levels of albumin serve as an indicator of malnutrition, they concurrently function as a biomarker for systemic inflammation [62–64]. Previous studies have demonstrated that inflammatory elements impede albumin synthesis, and oxidative stress can induce albumin denaturation, thereby contributing to a swift decline in serum albumin concentrations among individuals experiencing an inflammatory condition [65, 66]. Besides, LDH levels reflect tumor growth and invasiveness in cancer, as LDH is involved in the metabolism of pyruvate to lactic acid [67]. Several studies have reported that elevated LDH levels are predictive of poor prognosis in ICI-treated patients with various cancers [68, 69]. Considering that the ALI and GRIm score incorporate NLR and Alb, it is believed to provide an even stronger indication of resistance to ICI treatment in patients with advanced cancer.

In this investigation, we conducted an initial meta-analysis to validate the prognostic utility of the ALI and GRIm score in evaluating the responsiveness of cancer patients to ICI therapy. The ALI and GRIm score offer a multitude of advantages that render them suitable for routine clinical applications. Their accessibility, facile quantifiability, reproducibility, and comparatively economical nature make them highly amenable for assessment [70]. Consequently, owing to their firmly established influence on the nutritional and immune status of the host, as well as their impact on cancer, the ALI and GRIm score stand poised as valuable instruments for predicting the therapeutic outcomes of ICIs in cancer patients. Tailored and timely nutritional and immunological interventions have the potential to enhance the prognosis of individuals afflicted with cancer.

It is important to highlight that all studies included in our analysis were retrospective studies, which may introduce limitations in terms of statistical validity. Besides, the vast majority of studies included in this analysis were NSCLC and HCC, and the role of ALI and GRIm in other cancers remains to be further investigated. There are too few studies included in the analysis of HCC-GRIm, and more studies are needed for a comprehensive analysis. Therefore, there is a critical need for additional rigorous investigations with larger sample sizes, specifically multicenter prospective studies, to validate and enhance the robustness of our findings. The role of ALI and GRIm in predicting toxicities during immunotherapy should also be further explored in the future.

## Conclusion

We propose that the ALI and GRIm score represent robust prognostic indicators and should be integrated into the routine assessment of cancer patients. Such inclusion could prove invaluable for healthcare practitioners in adapting treatment regimens and promptly providing tailored nutritional support.

### Electronic supplementary material

Below is the link to the electronic supplementary material.

Supplementary Material 1

Supplementary Material 2

## Data Availability

No datasets were generated or analysed during the current study.
