# Supplementary material for: Association of the Advanced Lung Cancer Inflammation Index (ALI) and Gustave Roussy Immune (GRIm) score with immune checkpoint inhibitor efficacy in patients with gastrointestinal and lung cancer
Source: BMC Cancer. 2024 Apr 8;24:428. doi: 10.1186/s12885-024-12149-1 (PMC11000368; doi:10.1186/s12885-024-12149-1)
Supplement: Supplementary file 1 — Supplementary Material 1 [file 12885_2024_12149_MOESM1_ESM.docx]

Supplementary material 1. Pubmed search strategy

((((((Camrelizumab) OR (Sintilimab)) OR (Tislelizumab)) OR (Toripalimab)) OR (Envafolimab)) OR (((((((((((((((((((((((((((((((((((((((((((((((((anti-CTLA4 antibody) OR (anti-CTLA4 antibodies)) OR (Immune Checkpoint Inhibitors)) OR (Checkpoint Inhibitors, Immune)) OR (Immune Checkpoint Inhibitor)) OR (Checkpoint Inhibitor, Immune)) OR (Immune Checkpoint Blockers)) OR (Checkpoint Blockers, Immune)) OR (Immune Checkpoint Blockade)) OR (Checkpoint Blockade, Immune)) OR (Immune Checkpoint Inhibition)) OR (Checkpoint Inhibition, Immune)) OR (PD-L1 Inhibitors)) OR (PD L1 Inhibitors)) OR (PD-L1 Inhibitor)) OR (PD L1 Inhibitor)) OR (Programmed Death-Ligand 1 Inhibitors)) OR (Programmed Death Ligand 1 Inhibitors)) OR (PD-1-PD-L1 Blockade)) OR (Blockade, PD-1-PD-L1)) OR (PD 1 PD L1 Blockade)) OR (CTLA-4 Inhibitors)) OR (CTLA 4 Inhibitors)) OR (CTLA-4 Inhibitor)) OR (CTLA 4 Inhibitor)) OR (Cytotoxic T-Lymphocyte-Associated Protein 4 Inhibitors)) OR (Cytotoxic T Lymphocyte Associated Protein 4 Inhibitors)) OR (Cytotoxic T-Lymphocyte-Associated Protein 4 Inhibitor)) OR (Cytotoxic T Lymphocyte Associated Protein 4 Inhibitor)) OR (PD-1 Inhibitors)) OR (PD-1 Inhibitor)) OR (PD 1 Inhibitors)) OR (Inhibitor, PD-1)) OR (PD 1 Inhibitor)) OR (Programmed Cell Death Protein 1 Inhibitor)) OR (Programmed Cell Death Protein 1 Inhibitors)) OR (pembrolizumab)) OR (nivolumab)) OR (atezolizumab)) OR (ipilimumab)) OR (avelumab)) OR (tremelimumab)) OR (durvalumab)) OR (cemiplimab)) OR (anti-PD-1 antibodies)) OR (anti-PD-1 antibody)) OR (anti-PD-L1 antibody)) OR (anti-PD-L1 antibodies)) OR (Immune Checkpoint Inhibitors[MeSH Terms]))) AND ((("Gustave Roussy Immune Score") OR (GRIm)) OR ("advanced lung cancer inflammation index"))
