# Supplementary figures and images for: Association of the Advanced Lung Cancer Inflammation Index (ALI) and Gustave Roussy Immune (GRIm) score with immune checkpoint inhibitor efficacy in patients with gastrointestinal and lung cancer
Source: BMC Cancer. 2024 Apr 8;24:428. doi: 10.1186/s12885-024-12149-1 (PMC11000368; doi:10.1186/s12885-024-12149-1)

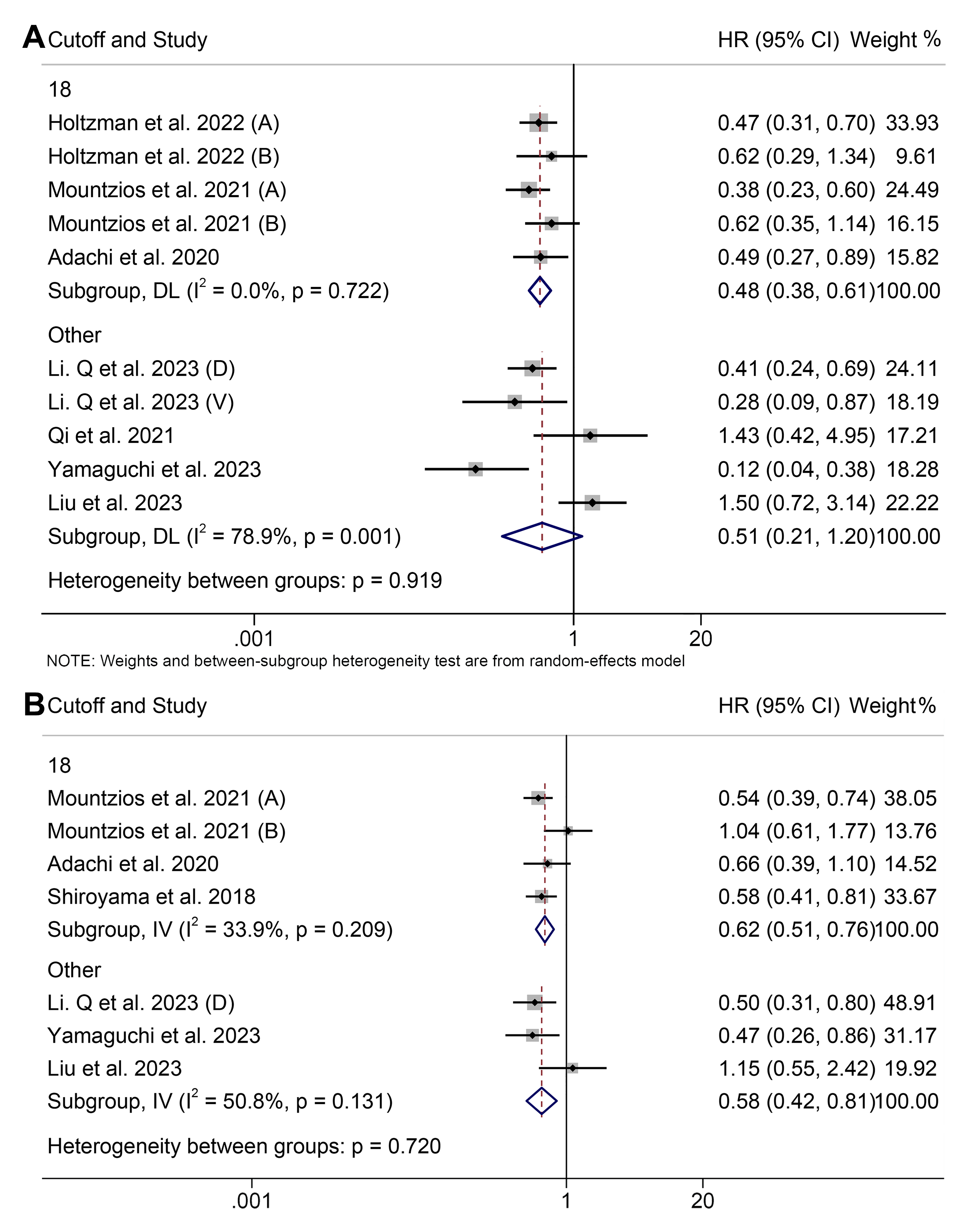

Supplement: Supplementary file 2 — Supplementary Material 2 [file 12885_2024_12149_MOESM2_ESM.tif]
